# Sinusoidal Endothelial Cell Progenitor Cells Promote Tumour Progression in Patients with Hepatocellular Carcinoma

**DOI:** 10.1155/2020/8819523

**Published:** 2020-11-28

**Authors:** Ya-xing Feng, Wei Li, Xu-dong Wen, Ning Zhang, Wei-hui Liu, Zhan-yu Yang

**Affiliations:** ^1^Department of Gastroenterology and Hepatology, Sichuan Academy of Medical Sciences & Sichuan Provincial People's Hospital, Chengdu, Sichuan Province, 610072, China; ^2^Department of Basic Medical Science, The General Hospital of Western Theater Command, Chengdu, Sichuan Province, 610083, China; ^3^Department of Gastroenterology and Hepatology, Chengdu First People's Hospital, Chengdu, Sichuan Province, 610016, China; ^4^Hepato-Pancreato-Biliary Surgery Centre, Chinese PLA General Hospital, Beijing 100039, China

## Abstract

**Objective:**

As sinusoidal endothelial cell progenitor cells (SEPCs) play a significant role in liver regeneration, it is necessary to elucidate whether SEPCs participate in tumour progression of hepatocellular carcinoma (HCC).

**Methods:**

A total of 45 patients with primary HCC who underwent liver resection were included in this study. The liver tumours were removed from the patients, and partial tissues were prepared to identify SEPCs through double staining of CD133/CD45 and CD133/CD31 at the same location. Blood samples were collected to examine liver function parameters and tumour markers. The demographics and clinicopathological characteristics of the patients were collected for correlation analysis with SEPCs.

**Results:**

SEPCs were observed in several blood vessels within the HCC nodules of all 45 patients, but no SEPCs were detected in the tumour-adjacent tissues. The number of SEPCs was correlated with the expression levels of HCC tumour markers *α*-fetoprotein (AFP) and CA199. There was a positive correlation between the expression of SEPC markers and diameter of HCC tumours in differently differentiated specimens (*P* < 0.01). The expression levels of SEPC markers were significantly higher in patients with poorly differentiated HCC than in patients with moderately and highly differentiated HCC (*P* < 0.05).

**Conclusions:**

SEPCs are closely associated with HCC progression; therefore, SEPCs may be considered potential prognostic and metastatic biomarkers and therapeutic candidates for HCC.

## 1. Introduction

Hepatocellular carcinoma (HCC) is the sixth most prevalent cancer worldwide and is the main cause of mortality due to cancer [1]. The high incidence of HCC in Asia and sub-Saharan Africa results from the high prevalence of hepatitis B virus (HBV) infection in these regions [2, 3]. Although surgical operation, radiotherapy, and immunotherapy are used for treating HCC, their curative effect on advanced HCC remains unsatisfactory owing to the side effects, drug resistance, and recurrence [4, 5]. Therefore, it is necessary to elucidate the molecular mechanisms of tumourigenicity and progression of HCC in order to obtain an early diagnosis, develop therapeutic strategies, and prevent tumour progression. To date, considerable studies have focused on tumour cells and related signalling pathways. Few studies have explored the effect of peritumour angiogenesis on malignant tumour formation and progression.

Angiogenesis is vital for malignant tumour formation, progression, and metastasis. Proliferation and migration of endothelial cells lead to angiogenesis, followed by the formation of new capillaries [6]. The hypoxic tumour microenvironment promotes excessive secretion of proangiogenic factors from neoplastic, stromal, and infiltrating immunocytes. Excessive proangiogenic factors stimulate abnormal angiogenesis, such as formation of blood vessels with structural abnormalities. Once tumour blood vessels are formed, they provide critical oxygen and nutrients to meet the metabolic requirements of tumour and support rapid tumour growth. Structurally abnormal and functional tumour vessels facilitate haematogenous metastasis, a hallmark of aggressive malignancies associated with poor survival rates [7]. Furthermore, these defective vessels are poorly perfused, limiting drug delivery to tumourous regions, thereby resulting in reduced efficacy of anticancer agents. Targeting vascular endothelial growth factor (VEGF) is one of the most common therapeutic strategies against tumour angiogenesis [8].

Angiogenesis requires a large number of sinusoidal endothelial cells, and sinusoidal endothelial cell progenitor cells (SEPCs), the progenitors of sinusoidal endothelial cells, have been found to be the seed cells of angiogenesis [9, 10]. SEPCs are triple positive for the progenitor cell marker CD133, the endothelial cell marker CD31, and the haematopoietic cell marker CD45 [11–13]. Numerous studies have indicated that engraftment of a substantial number of SEPCs observed after liver injury or partial hepatectomy involves VEGF-stromal cell-derived factor 1 (SDF1) signalling [14–18]. In addition, VEGF has been reported to stimulate SEPCs to secrete hepatocyte growth factor (HGF) [15]. Although angiogenesis, regulated by several key factors, including VEGF, has been shown to correlate with HCC progression, the roles of SEPCs in angiogenesis during HCC development have not yet been investigated. Herein, we aimed to explore the potential relationship between SEPCs and HCC progression.

## 2. Patients and Methods

### 2.1. Patients and Samples

A total of 45 patients with histologically confirmed primary HCC, who underwent surgical resection at the Sichuan Academy of Medical Sciences and Sichuan Provincial People's Hospital between January 2017 and December 2019, were consecutively enrolled in the present study. None of the patients had other malignancies or a history of drinking for at least five years. In addition, these patients were diagnosed with the disease for the first time, and they did not receive radiotherapy, chemotherapy, or immunotherapy. Patients were assessed based on clinical examination, laboratory parameters, ultrasound, CT scans, and MRI imaging.

Blood and tissue samples and patient data were provided by the Sichuan Academy of Medical Sciences and Sichuan Provincial People's Hospital. All samples were fully anonymised except for sex and age. Written informed consent was obtained from all patients, and the study was approved by the institutional review board of the Sichuan Academy of Medical Sciences and Sichuan Provincial People's Hospital.

This study was performed in accordance with the clinical study protocols and principles of the Declaration of Helsinki (modified 2000) and was approved by the Research Care and Ethics Committee of our institution (No. SPPHCT2017–MED-10).

### 2.2. Clinical Features

Clinical features were obtained from the enrolled patients, including demographic characteristics (age, sex, BMI, ethnic group, history of smoking, and history of drinking), clinicopathologic features (HBV surface antigen, pathological grade, and metastasis), laboratory parameters (blood routine test, total protein, albumin, total bilirubin, direct bilirubin, indirect bilirubin, ALT, and AST), tumour markers (AFP, CEA, CA-199, and CA-125), and tumour diameter (the largest diameter of tumour).

### 2.3. Blood Sampling

Blood samples from the patients with HCC were centrifuged at 1500 g/min for 10 min at 4°C, followed by centrifugation at 2000 g/min for 3 min at 4°C. Thereafter, the serous samples were stored at -80°C. Routine laboratory parameters were recorded at the Department of Clinical Laboratory in the Sichuan Academy of Medical Sciences and Sichuan Provincial People's Hospital.

### 2.4. Haematoxylin-Eosin (HE) Staining and Immunofluorescence Staining

A total of 45 pairs of tumourous and adjacent nontumourous liver tissues were obtained from surgically resected HCC specimens. Samples were then formaldehyde-fixed, paraffin-embedded, and sectioned into 5 *μ*m slices. The sections of 45 tumourous liver tissues were dewaxed in xylene, followed by HE staining and microscopic observation, to determine the differentiation grades of HCC. Immunohistochemistry staining scores of HCC differentiation in liver tissues were assessed using a semiquantitative method by two pathologists who were blinded to the clinical data prior to the assessment. Immunofluorescence staining was performed on the sections of 45 pairs of tumourous and adjacent nontumourous liver tissues using an immunofluorescence staining kit. Endogenous avidin and biotin were blocked using an avidin-biotin kit (Vector Labs) following the protocol. Primary antibodies including anti-CD133 (cat. BF0403, Affinity Biosciences, OH, USA), anti-CD45 (cat. DF2912, Affinity Biosciences, OH, USA), and anti-CD31 (cat. AF6191, Affinity Biosciences, OH, USA) were used in this study. All the primary antibodies were diluted 1 : 250 and applied to the coverslips overnight at 4°C. The primary antibody was recognised by the fluorescent secondary antibodies (cat. ab6785 and cat. ab97077, Abcam Biochemicals, MA, USA) and was visualised using a fluorescence inverse microscope (Olympus X81). Four views of each section were randomly recorded, with three sections obtained from one sample. The positive staining signals in HCC tissues were quantitated using Image-Pro Plus software. The sections were observed using an Olympus microscope at 10 × 10 magnification. The target specimens were double-stained for CD133/CD45 and CD133/CD31.

### 2.5. Statistical Analysis

Continuous variables are shown as means ± standard deviation, and categorical variables are reported as frequencies and percentages. Differences in the expression levels of SEPC markers between different patient cohorts were determined using ANOVA. *P* < 0.05 was considered statistically significant. Statistical analyses were performed using SPSS (version 22.0, IBM, New York, USA). Figures were prepared using GraphPad Prism version 6.0 (GraphPad Software, San Diego, California, USA).

## 3. Results

### 3.1. Demographic Characteristics of Patients and Histopathology

A total of 45 patients with HCC were reviewed at the Sichuan Academy of Medical Sciences and Sichuan Provincial People's Hospital from January 2017 to December 2019, all of whom met the inclusion criteria. The demographics and clinicopathological parameters of the enrolled 45 patients, including age, gender, ethnic group, pathological grade, metastasis, and presence of HBV, are shown in Table 1. All 45 patients were of Han nationality, and they were diagnosed with primary HCC based on pathological examination for the first time and did not have other malignancies. The proportion of patients who were male and aged >50 years was 55.6% and 91.1%, respectively.

**Figure 1:**
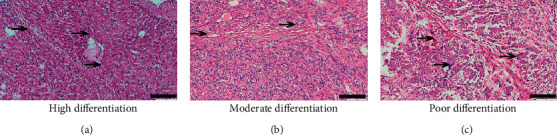
Degrees of differentiation in hepatocellular carcinoma (HCC). A typical image of highly differentiated HCC, in which cells with an increased nucleocytoplasmic ratio were arranged along the hepatic trabecula, accompanied by changes in the alveolar structure and fat content (arrows, a). Moderately differentiated HCC with clear nucleolus cells that were rich in cytoplasm and were arranged into small trabecular structures or cell cords (arrows, b). Poorly differentiated HCC cells with a significantly increased nucleocytoplasmic ratio and obvious pleomorphism (arrows, c). Scale bars: 100 *μ*m.

HE staining results suggested that differently differentiated HCC lesions were detected in 45 patients. Typical cases of HCC with poor differentiation, moderate differentiation, and high differentiation are shown in Figure 1. In highly differentiated HCC, most of the cancer cells with an increased nucleocytoplasmic ratio were arranged along the hepatic trabecula and were accompanied by changes in the alveolar structure and fat content (Figure 1(a)). In moderately differentiated HCC, cells with a clear nucleolus and rich cytoplasm were arranged into small trabecular structures or cell cords (Figure 1(b)). In poorly differentiated HCC, the nucleocytoplasmic ratio of cancer cells increased significantly, and the tumour cells showed obvious pleomorphism, variation in size, and distinct shape (Figure 1(c)).

Additionally, the majority (86.7%, 39/45) of HCC patients were HBV positive, and there was no pathologic difference between HBV positive and negative cases. Pathological grade including high differentiation, moderate differentiation, and low differentiation accounted for 20% (9/45), 20% (9/45), and 60% (27/45) patients, respectively. Among these patients, 11 (24.4%) did not have metastases, while 34 (75.6%) had metastases. Focal liver lesions were located in the right liver lobe or the left three-lobe liver. Correspondingly, these 45 patients underwent right hepatectomy or left trisegmentectomy according to the location of the focal liver lesion and did not receive radiotherapy, chemotherapy, or immunotherapy.

### 3.2. Localisation of SEPCs in HCC Patients

**Figure 2:**
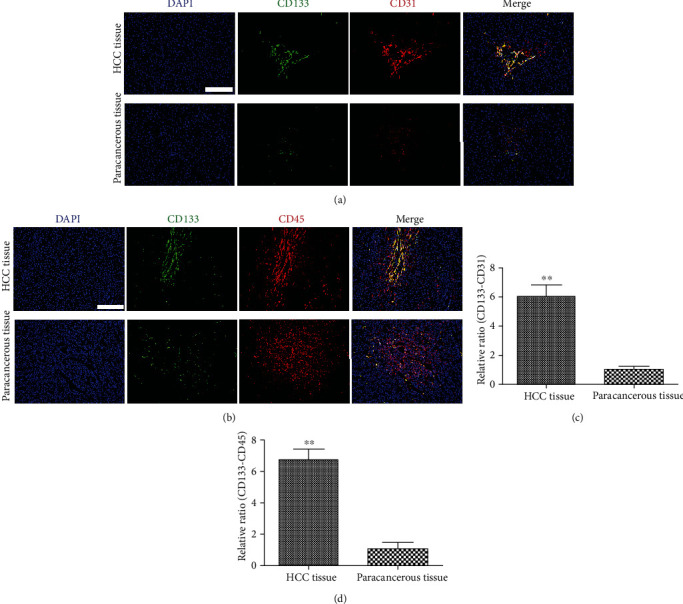
Localisation of sinusoidal endothelial cell progenitor cells (SEPCs) in the liver tissues of HCC patients. Representative immunofluorescence images of SEPC markers (CD133, CD31, and CD45) in liver tissues from HCC patients found abundantly around the blood vessels in tumour tissues. Few SEPCs were observed in corresponding tumour-adjacent tissues (a, b). Magnification: 100x. Expression levels of SEPC markers (CD133, CD31, and CD45) were significantly higher in tumourous tissues than in adjacent nontumourous liver tissues (c, d). ^∗∗^*P* < 0.01. Scale bars: 100 *μ*m.

SEPCs that expressed CD31, CD45, and CD133 were considered progenitors of liver sinusoidal endothelial cells (LSECs) [11, 12]. Therefore, we performed double staining for CD133/CD45 and CD133/CD31 at the same location to prove that the staining was triple positive to determine the phenotypic signature for SEPCs. SEPCs were found in several blood vessels of tumour nodules from all 45 patients, while no SEPC was detected in the tumour-adjacent tissues (Figure 2). Significantly more SEPCs were present in tumourous tissues than in adjacent nontumourous liver tissues (*P* < 0.01) (Figure 2).

### 3.3. Liver Functional Data

**Figure 3:**
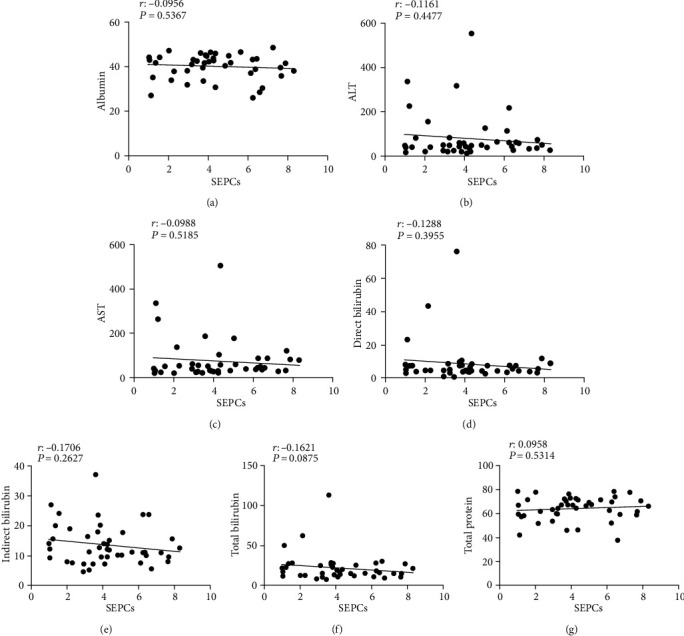
Spearman correlation between SEPCs and liver function parameters. There was no significant correlation between SEPC expression and albumin (a), alanine aminotransferase (b), glutamic-oxalacetic transaminase (c), direct bilirubin (d), indirect bilirubin (e), total bilirubin (f), and total protein (g) within the 45 HCC patients.

Table 2 shows the liver functional data, including total protein, albumin, direct bilirubin, indirect bilirubin, ALT, and AST. Total protein, albumin, direct bilirubin, indirect bilirubin, and ALT levels were normal in most cases, and AST exhibited an approximately equal ratio of high and normal cases (23 versus 22 cases). A linear regression analysis revealed that there was no correlation between the number of SEPCs and total protein, albumin, direct bilirubin, indirect bilirubin, ALT, and AST levels (Figure 3).

### 3.4. Tumour Markers

**Figure 4:**
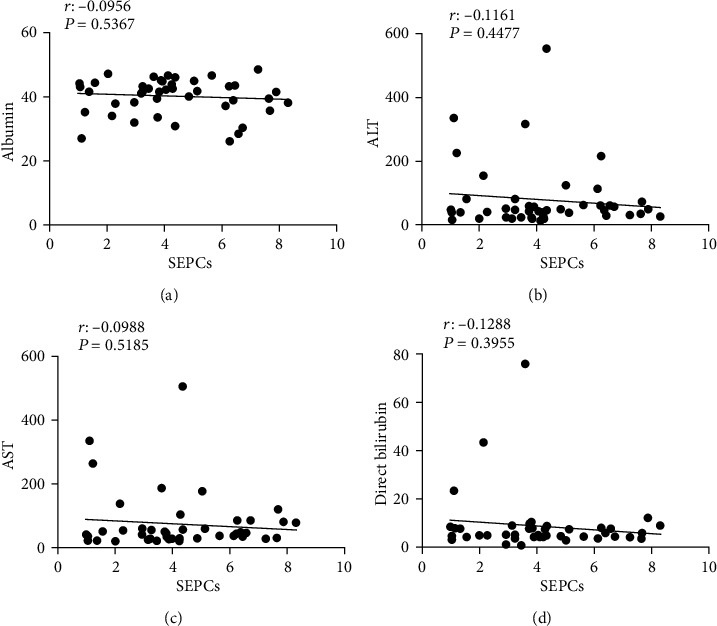
Spearman correlation between SEPCs and tumour-related markers. There were positive correlations between SEPC expression and alpha-fetoprotein (a) and CA199 (b) levels, but there was no correlation between SEPC expression and carcinoembryonic antigen (c) and CA125 levels (d).

As shown in Table 3, levels of HCC tumour markers, including AFP, CA199, CEA, and CA125, were determined. Most patients had high levels of AFP, while CA199, CEA, and CA125 levels were normal in most HCC patients. Furthermore, linear regression analysis indicated that there was a strong positive correlation between SEPC marker expression and AFP or CA199 levels (Figures 4(a) and 4(b)). However, there was no correlation between the expression of SEPC markers and CEA and CA125 levels (Figures 4(c) and 4(d)).

### 3.5. Relationship between SEPCs and the Size of HCC

**Figure 5:**
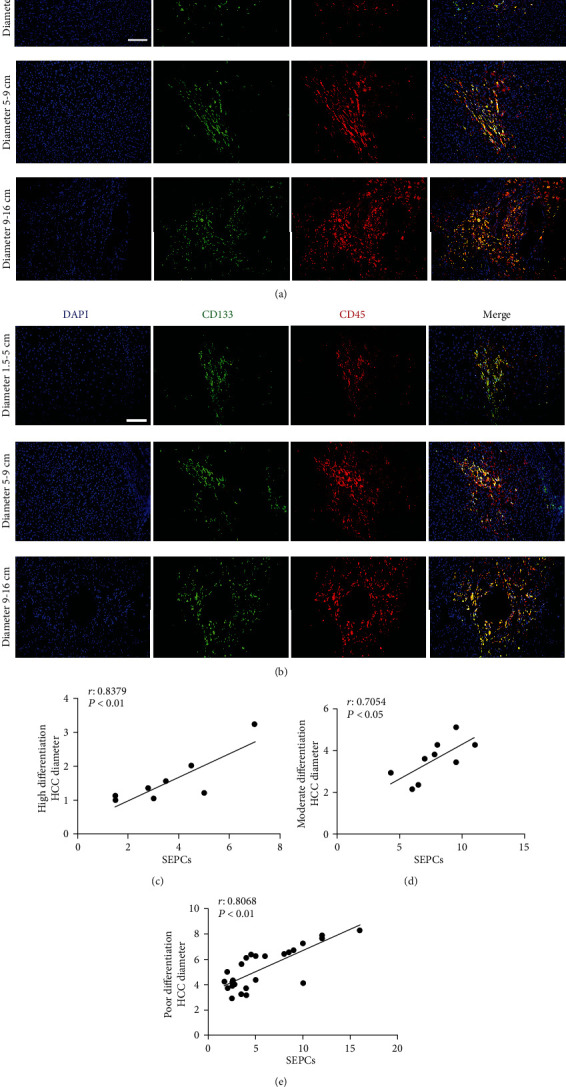
SEPC expression in HCC lesions of different sizes and correlations of SEPC expression with sizes of tumours of various differentiation grades. Representative immunofluorescence images of SEPC markers (CD133, CD31, and CD45) in differently sized HCC lesions (a, b). Magnification: 100x. There was a significant correlation between SEPC expression and tumour sizes in highly differentiated (c), moderately differentiated (d), and poorly differentiated (e) specimens. Scale bars: 100 *μ*m.

The diameter of HCC tumours (the largest diameter of the tumour) in highly, moderately, and poorly differentiated specimens was measured to explore the relationship between SEPCs and the size of HCC lesions in the differentiated cases. According to the diameter of tumours, samples were divided into three groups, i.e., 1.5–5 cm, 5–9 cm, and 9–16 cm groups. We then observed the expression of SEPC markers in each HCC specimen (Figures 5(a) and 5(b)). Linear regression analysis revealed that there was a strong positive correlation between SEPC content and HCC tumour diameter in highly, moderately, and poorly differentiated specimens (*P* < 0.01) (Figure 5(c)).

### 3.6. Relationship between SEPC Content and Differentiation Grades of HCC

**Figure 6:**
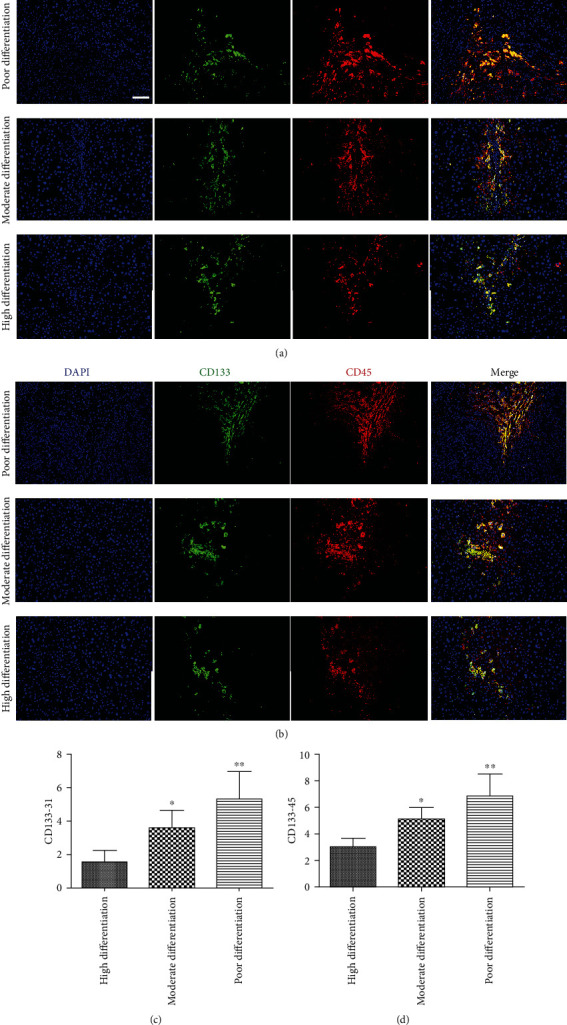
SEPC expression in HCC lesions of different differentiation grades. Representative immunofluorescence images of SEPC markers (CD133, CD31, and CD45) in different differentiations of HCC specimens (a, b). Magnification: 100x. The number of SEPCs in HCC specimens with different differentiations was analysed. Significant differences in SEPC expression were found between highly, moderately, and poorly differentiated groups in the CD133-CD31 group (c), as well as in the CD133-CD45 group (d). ^∗^*P* < 0.05, ^∗∗^*P* < 0.01. Scale bars: 100 *μ*m.

In order to explore the relationship between SEPCs and the differentiation of HCC, we investigated the number of SEPCs in highly differentiated, moderately differentiated, and poorly differentiated specimens. The SEPC number was significantly higher in poorly differentiated HCC than in moderately and highly differentiated HCCs (*P* < 0.01) (Figure 6(b)). Compared with highly differentiated HCC, the expression of SEPC markers in moderately differentiated HCC was also statistically different (*P* < 0.05).

## 4. Discussion

HCC is the third leading cause of cancer-related mortality worldwide, mainly due to the advanced stage of HCC at the time of initial diagnosis [19]. The high rate of recurrence and resistance of HCC to conventional therapy is caused by the high angiogenic and metastatic potential of HCC cells; the rate of recurrence is as high as 70% following conventional treatments, such as chemotherapy, arterial embolisation, surgical resection, and radiofrequency ablation [20–22]. The present study mainly focused on HCC cancer cells, including metabolism of cancer cells [21, 23] and the high degree of molecular heterogeneity in liver tumours [24]. Few studies have explored the effect of the peritumour environment, such as angiogenesis, on malignant tumour formation and progression.

Angiogenesis, an important feature of cancer, is induced early during the multistage development of cancers [25, 26]. High levels of angiogenesis-related factors are significantly associated with rapid cancer recurrence and poor survival [27, 28]. Blocking synthesis and secretion of these growth factors would greatly impair tumour cell proliferation, metastasis, and angiogenesis [29–32]. However, the underlying mechanisms are not fully understood, and few studies have been conducted to explore the effect of peritumour angiogenesis and vessel released factors on tumour diagnosis, formation, development, and metastasis. Hence, the discovery of novel diagnostic and therapeutic targets for angiogenesis is crucial for patients with HCC.

A small number of SEPCs are progenitors of LSECs that exist in normal liver tissue and express CD31, CD45, and CD133. Previous studies have indicated that bone marrow- (BM-) derived sinusoidal endothelial cell progenitor cells (BM SEPCs) drive the liver regeneration process. These BM SEPCs repair the lost or injured LSECs, leading to engraftment of a substantial number of BM SEPCs and BM-derived LSECs after injury or partial hepatectomy [14, 16]. Recruitment of BM SEPCs to the liver occurs through activated signalling of VEGF-SDF1, and BM SEPCs lead to a significant increase in the LSEC-related HGF level after injury, further promoting hepatocyte proliferation and normal liver regeneration [8, 15]. Previous studies have indicated that hepatic VEGF regulates the recruitment of BM SEPCs to the injured liver to help repair damage [15], and the influx of BM SEPCs leads to a significant increase in HGF levels after injury and promoted hepatocyte proliferation and normal liver regeneration [11]. However, no studies have been conducted to explore whether SEPCs are related to HCC progression. This study revealed the presence of SEPCs in HCC, which could be detected in vascular-rich areas of the tumour tissues. In addition, we also demonstrated that the contents of SEPCs were related to the expression of AFP, tumour size, and differentiation grade of HCC. Together with previous studies, we speculated that SEPCs might be crucial in the progression of HCC through the secretion of factors and tumour angiogenesis effects.

HCC is difficult to diagnose early and to treat promptly and effectively due to the lack of typical symptoms at an early stage. At present, AFP is the most common serological test used for screening and diagnosis of HCC as well as for surveillance after treatment. However, there were serious limitations with AFP, such as low sensitivity [33], false-negatives, and false-positives, owing to conditions such as pregnancy and certain gastrointestinal tumours [34, 35]. In this study, SEPC levels demonstrated a positive correlation with AFP levels, indicating that SEPCs could be a potential target in HCC diagnosis by acting as a complementary tumour marker. Comparison between AFP and SEPC in the diagnosis of liver cancer, with pathological diagnosis as the gold standard, needs to be conducted in the future to explore whether SEPCs perform better than AFP in HCC. Future studies could investigate the potential roles of factors secreted by SEPCs as complementary tumour markers in diagnosis.

In our study, we also explored the relationship between SEPCs and tumour size of HCC. Since the tumour size varied greatly even in the same differentiation grade, we measured the diameter of HCC tumours (the largest diameter of the tumour) in highly, moderately, and poorly differentiated specimens separately. Linear regression analysis revealed that there was a positive correlation between SEPC content and HCC tumour diameter in differently differentiated specimens (*P* < 0.01). Hepatocarcinogenesis was determined by an unbalanced angiogenesis process with augmented production of proangiogenic factors (drivers of vessel growth and maturation) by tumour cells and adjacent cells, including VEGF, platelet-derived growth factor [36–38]. Tumour size was closely related to tumour angiogenesis, which provided the nutrition-carrying blood for tumour growth. In this study, those SEPCs distributed along the blood vessels in tumour nodules were demonstrated, and the positive correlation between SEPC numbers and HCC tumour diameter was confirmed. The above findings indicated that SEPCs are related to tumour angiogenesis. In fact, most of the currently approved treatments for advanced HCC in first- and second-line settings target angiogenic pathways. Of the known or potential angiogenic pathways in tumours, the VEGF/VEGF receptor (VEGFR) signalling pathway has been validated as a drug target in HCC [39]. Therefore, SEPCs might serve as a potential antiangiogenic therapeutic target for HCC.

Furthermore, results from this study revealed that the SEPC content in patients with poorly differentiated HCC was significantly higher than that in patients with moderately and highly differentiated HCC (*P* < 0.01), indicating that SEPCs could be a risk factor for prognosis and metastasis. This is also the first study demonstrating that SEPCs are related to the differentiation of HCC. Differentiation is closely related to tumour invasion and metastasis in various tumours, including HCC. Thus, SEPCs could be used as a potential prognostic marker for distinguishing poorly differentiated HCC from well-differentiated HCC.

Considering that the contents of SEPCs were associated with tumour marker levels, tumour size, and differentiation grades of HCC, SEPCs might be applied as a marker for HCC diagnosis, a risk stratification index to predict the prognosis and metastasis of HCC, and a therapeutic target for HCC. However, the sample volume and the follow-up data were limited, and more samples and follow-up data are required to validate our conclusion in further studies. In addition, the underlying mechanisms regarding the roles of SEPCs in tumour angiogenesis and progression of HCC remain to be established.

## Figures and Tables

**Table 1:** Demographics and clinicopathological characteristics of HCC patients.

Variable	Classification	Number
Age (year)	≥50	25
	<50	20
Gender	Male	41
	Female	4
Hepatitis B infection	Yes	39
	No	6
Differentiation grade	High	9
	Moderate	9
	Poor	27
Metastasis	Yes	34
	No	11

**Table 2:** Liver function indexes in patients with hepatocellular carcinoma.

Variable	Classification	Number
Total protein (g/L)	Normal (65-85)	27
	Low (≤65)	18
Albumin (g/L)	Normal (40-55)	31
	Low (≤40)	14
Total bilirubin (*μ*mol/L)	Normal [5–28]	38
	High (≥28)	7
Direct bilirubin (*μ*mol/L)	Normal (0-7)	26
	High (≥7)	19
Indirect bilirubin (*μ*mol/L)	Normal [3–23]	39
	High (≥23)	6
ALT (U/L)	Normal (9-60)	30
	High (≥60)	15
AST (U/L)	Normal (15-45)	22
	High (≥45)	23

Abbreviation: ALT = glutamate-pyruvate transaminase; AST = glutamic-oxalacetic transaminase.

**Table 3:** The expression levels of tumour markers in hepatocellular carcinoma patients.

Variable	Classification	Number
AFP (ng/mL)	Normal (0-9)	4
	High (≥9)	41
CA-199 (U/mL)	Normal (0-35)	30
	High (≥35)	15
CEA (ng/mL)	Normal (0-10)	35
	High (≥10)	10
CA-125 (U/mL)	Normal (0-35)	33
	High (≥35)	12

Abbreviation: AFP = *α*-fetoprotein; CEA = carcinoembryonic antigen.

## Data Availability

Data (blood indexes, immunofluorescence, and immunohistochemical results) that are directly related to the acquired results are available. For more detailed information, the readers can consult Ya-xing Feng (email: 124503329@qq.com, telephone number: 0086-17780961245). Written informed consent was obtained from all patients, and the study was approved by the institutional review board of Sichuan Academy of Medical Sciences and Sichuan Provincial People's Hospital. Data concerning admission documents are not freely available due to the patients' privacy.

## Acronyms

AFP: *α*-Fetoprotein
BM SEPCs: Bone marrow- (BM-) derived sinusoidal endothelial cell progenitor cells
HBV: Hepatitis B virus
HCC: Hepatocellular carcinoma
HGF: Hepatocyte growth factor
LSECs: Liver sinusoidal endothelial cells
SEPCs: Sinusoidal endothelial cell progenitor cells
VEGF: Vascular endothelial growth factor.
